# Brand Sharing and New Drinking Occasions: A Content Analysis of How Alcohol Brands in Australia and Aotearoa New Zealand Promote Zero Alcohol Products

**DOI:** 10.1111/dar.70148

**Published:** 2026-04-13

**Authors:** Danica Keric, Fraser Edwardes, Julia Stafford, Nathan J. Harrison, Joelie Mandzufas, Ashlea Bartram, Simone Pettigrew

**Affiliations:** ^1^ Cancer Council Western Australia Perth Australia; ^2^ Flinders Health and Medical Research Institute, College of Medicine and Public Health Flinders University Adelaide Australia; ^3^ National Centre for Education and Training on Addiction, Flinders Health and Medical Research Institute, College of Medicine and Public Health Flinders University Adelaide Australia; ^4^ The George Institute for Global Health UNSW Sydney Sydney Australia

**Keywords:** advertising, alcohol industry, alcohol policy, NoLo, zero alcohol

## Abstract

**Introduction:**

The rapid expansion of zero alcohol products (ZAP) and associated marketing is of increasing public health interest. Investigating marketing approaches can provide insights to inform policy responses to this emerging issue.

**Methods:**

We conducted a content analysis of marketing campaigns by alcohol‐branded ZAPs in Australia and Aotearoa New Zealand (NZ). We used publicly available information to systematically: (i) identify alcohol‐branded ZAPs available in each country; (ii) collect information about marketing campaigns for each product; (iii) collect marketing assets; and (iv) identify campaign marketing strategies. The coding frame incorporated a combination of deductive (based on existing literature) and inductive codes.

**Results:**

We identified 13 alcohol‐branded ZAPs in Australia and six in NZ that were promoted in 20 and 10 marketing campaigns, respectively, between 2018 and 2024. Mateship/friendship was the most common strategy, present in 80% of campaigns. Almost two‐thirds of all campaigns (63%) promoted the product in the context of new drinking occasions (e.g., at the gym, while driving). Benefits of ZAP use included providing choice of a viable alcohol alternative (73% of campaigns), taste (47%), fitness/health (27%) and productivity (23%).

**Discussion and Conclusions:**

This study provides evidence from Australia and NZ of alcohol‐branded ZAP marketing campaigns promoting novel drinking occasions, adding to concerns raised by public health professionals. Stronger community protections for alcohol marketing, including marketing of ZAPs that share branding with alcoholic products, are warranted. Established alcohol brands appear to be using ZAP marketing campaigns to also promote alcohol branding in new drinking contexts.

## Key Points Summary

- Analysing zero alcohol product marketing campaigns can help inform policy responses to concerns about exposure to alcohol marketing.
- Marketing campaigns for zero alcohol products that share branding with alcoholic products tend to promote alcohol branding in new drinking contexts such as while exercising, driving or operating heavy machinery.
- Stronger community protections for alcohol marketing, including marketing for zero alcohol products that share branding with alcoholic products, are warranted.

## 1 Introduction

Replacing alcoholic products with products containing little or no alcohol may be a potential alternative for adults seeking to reduce their alcohol intake [1]. However, emerging research suggests that companies often promote zero or very low alcohol products (referred to as zero alcohol products or ZAPs for the remainder of the paper) as products to drink in addition to rather than as a replacement for alcoholic products [2, 3]. This is further evidenced by alcohol industry communications; in announcing new ZAP marketing campaigns, alcohol advertising executives have reported an intent to compete with the (non‐alcoholic) soft drink market and expand opportunities for people to consume their products [4, 5, 6, 7]. For example, an alcohol company executive described a zero alcohol beer product as ‘giving people the freedom to enjoy their favourite drink in places where beer is not usually consumed’ [7]. While there is no global agreement on a definition, we define zero and no alcohol products as those containing 0.5% alcohol by volume (ABV) or less. By comparison, ‘NoLos’ are generally accepted to mean no more than 1.15% ABV [8].

ZAPs that share branding with established alcohol brands dominate the zero alcohol product market [1]. For example, in a sample of the best‐selling ZAPs in the United Kingdom, the majority of beer (94%) and ready to drink products (77%) had an alcohol parent brand [9]. Marketing associated with ZAPs is an emerging issue that presents new and unique challenges for public health practitioners and policymakers [1]. Experimental research has shown that ZAPs, particularly those from alcohol brands, make adolescents think of alcohol [10]. In addition, adolescents who see and like advertisements for ZAPs featuring an alcohol brand have more favourable attitudes towards and stronger intentions to consume alcoholic products from that brand [11].

Exposure to alcohol marketing is associated with alcohol use among children and young people [12]. The more young people are exposed to alcohol advertising, the greater the likelihood that they will start to use alcohol or drink more if they are already using alcohol [13]. There is also evidence of a small but consistent association of exposure to advertising with alcohol use at a population level [14, 15, 16, 17], and research shows an adverse effect of alcohol marketing on people experiencing alcohol dependence [18]. Recognising the impacts of alcohol advertising, the World Health Organization recommends governments enact and enforce bans or comprehensive restrictions on exposure to alcohol advertising as one of three ‘best buys’ to reduce the harmful use of alcohol [19]. In addition, given the emerging evidence about advertising of ZAPs being associated with alcohol attitudes and intentions, the regulation of ZAP advertising is also recommended [1].

The context of the present study is in Australia and Aotearoa New Zealand (NZ), where alcohol use is high by world standards (10.1 and 9.9 L, respectively, compared to 5.5 L globally in 2019) and causes significant harms [20]. For example, 6711 deaths in Australia and 1248 deaths in NZ were attributable to alcohol consumption in 2019 [20]. Both countries use voluntary and co‐regulatory approaches to alcohol advertising that appear to be similar and have been demonstrated to be ineffective [21], despite recommended reforms that would prioritise government‐led frameworks [22, 23, 24]. Some products, campaigns and/or assets may only be visible in specific regions. However, given that alcohol is increasingly marketed across national borders, it is possible that exposure to alcohol marketing is similar in both countries [25]. Alcohol industry analysts report substantial increases in availability of and demand for ZAPs in Australia and NZ [26, 27]. The Alcohol Beverages Advertising Code Responsible Alcohol Marketing Code sets voluntary guidelines for alcohol marketing in Australia and extends to non‐alcohol products (< 0.5% ABV) sharing branding with alcoholic products, known as ‘alcohol alternatives’ [28]. Data are lacking on the marketing campaign strategies used to promote ZAPs that share branding with alcoholic products in Australia and NZ, and this information is needed to inform future policy direction. To assist in addressing this evidence deficit, the aim of the present study was to identify and describe key strategies used in alcohol‐branded, video‐led ZAP marketing campaigns in Australia and NZ.

## 2 Methods

### 2.1 Study Design

We conducted a content analysis of marketing campaigns, using publicly available information on websites and social media. We: (i) identified alcohol‐branded zero alcohol products available in Australia and NZ; (ii) collected information about video‐led marketing campaigns for these products in each of these countries; (iii) collected the marketing assets; and (iv) identified strategies used in alcohol‐branded ZAP marketing campaigns. As this research did not meet the criteria for human research, ethics approval was not sought.

### 2.2 Sample Identification

We used guides to the alcohol industry in Australia [29] and NZ [30] to identify major alcohol companies and their leading brands. We searched the alcohol company and brand websites to identify alcohol‐branded ZAPs and conducted targeted Google searches of alcohol brand names along with the terms ‘zero alcohol’ or ‘non‐alcoholic’ (the first two pages of results were reviewed). To ensure a comprehensive list of ZAPs that shared branding with an alcoholic product, we also searched major alcohol retailer websites in each country. We were open to including products up to 1.15% ABV. Based on this information, we prepared a list of ZAPs manufactured or distributed by alcohol companies in Australia and NZ.

### 2.3 Data Collection

We recorded data pertaining to video‐led marketing campaigns for each identified zero alcohol product in each country, using a five‐step approach to create a comprehensive list of all campaigns and marketing assets. First, we searched the brand and company websites for links to marketing campaigns or assets and links to social media pages including Instagram, Facebook, YouTube, LinkedIn and X (Twitter). TikTok was excluded owing to the platform's self‐imposed restrictions on alcohol marketing in Australia at the time of project planning and data collection [31]. Second, we searched the brand and company social media pages for marketing campaigns promoting the zero alcohol product from the date of the product launch. A researcher aged over 18 years (F.E.) was logged into a personal account for each social media platform to ensure access to age‐gated campaigns or marketing assets. Third, we performed targeted Google searches using the product and brand name and reviewed the first two pages of results for additional webpages, videos, imagery and news articles. Fourth, we performed a search on the Meta Ad Library for marketing campaigns; we searched by advertiser, then brand/company name, selecting the appropriate regional account where relevant. To capture print assets, in the final step we searched all issues of known alcohol industry trade magazines from 2018, the year the earliest campaign was in market (*n* = 3 titles in Australia, *n* = 2 titles in NZ).

Campaigns from alcohol producers or distributors were included if they had at least one video component, were executed on multiple media channels (e.g., TV, radio, out‐of‐home, digital) and were either currently or previously in market in Australia or NZ. We excluded adhoc advertisements or social media posts, social media influencer posts and alcohol retailer promotions for ZAPs. We extracted details about each marketing campaign, including the parent alcohol company name, brand name, campaign name, launch date and media channels (Table 1). Each campaign comprised at least one video advertisement and may have included other marketing assets such as static or digital advertisements, radio advertisements and social media advertisements. Details of each marketing asset were recorded including the link to their location online, and each asset was downloaded to a local server to enable repeated viewing and coding. The data collection period was July–September 2024.

**TABLE 1:** Proportion of campaigns assigned to each category and code, overall and by country.

Code	Australia campaigns	NZ campaigns	Total
	(*n* = 20), %	(*n* = 10), %	(*n* = 30), %
Occasion framing	90	90	90
Established occasions^a^ (total)	75	70	73
In bar or pub	60	40	53
Partying	35	10	27
Special occasion	25	0	17
Watching sport	5	10	7
Novel occasions^b^ (total)	65	60	63
Novel occasions: context expansion	65	60	63
On or around water	35	40	37
Driving	50	10	36
Adrenaline activities	40	10	30
Exercising or playing sport	25	10	20
Work	20	10	17
Novel occasions: temporal expansion	30	20	27
Abstinence periods (e.g., Dry July or Ocsober)	15	0	10
While pregnant	5	0	3
Benefits of consumption mentioned or implied	80	100	93
Choice of viable alcohol alternative	75	70	73
Taste/refreshing	45	50	47
Health benefits	30	20	27
Quality product	20	40	27
Productivity/control	20	30	23
Being a ‘better’ person	20	20	20
Lifestyle, identity and value signifiers	95	80	90
Mateship/friendship	90	60	80
Urban lifestyle	60	40	53
Nature/conservation	50	50	50
Humour	55	30	47
Sport	45	30	40
Exclusivity and expertise	30	10	23
Masculinity/manliness	15	20	17
Future	20	0	13
Family	20	0	13
Femininity/gender	10	10	10
Heritage	10	10	10
First Nations peoples	0	10	3
Sexual attraction	5	0	3
Consumption cues	60	30	50
Reward or relaxation	50	30	43
Drinking to excess/inconsistent with community standards	35	10	27
How to drink/cocktails/recipes	5	0	3

^a^ ‘Established occasion’ was coded when there was an implicit or explicit reference to ZAP being a replacement for an alcoholic product, or used in a context that has traditionally been an occasion where the use of alcohol is seen as appropriate.

^b^ ‘Novel occasion’ refers to a context or occasion that expands current drinking contexts, including contexts or occasions not typically associated with alcoholic products, not in line with how alcoholic products are generally promoted, contrary to accepted public health standards or not socially or legally acceptable occasions to use alcohol.

### 2.4 Coding Frame Development and Coding Methods

The coding frame incorporated a combination of deductive and inductive codes. The initial frame was based on codes used in reviews of similar campaigns [2, 32, 33]. To ensure a common interpretation of each category and code, two coders (D.K. and F.E.) discussed the coding frame and data dictionary (see Table S2), and agreed on a structured coding procedure. Each marketing asset was assessed against each code with a binary response of Yes (present) or No (absent) recorded in an Excel spreadsheet. The two coders jointly coded the first 20 marketing assets (from multiple campaigns) before coding the remaining assets independently. The coders met after assessing each set of 20 marketing assets to review coding consistency, discuss discrepancies and resolve disagreements until agreement was achieved. Intercoder reliability was high, with simple proportion agreement at 93% prior to discrepancies being resolved. When new codes were identified, they were added to each coder's workbook, with the definition added to the data dictionary. Each new code was then applied to marketing assets coded before its identification and those assets yet to be coded, to ensure all assets were assessed against each code. The final coding frame comprised 38 lower‐level codes organised under five categories (see Tables 1 and S2).

### 2.5 Statistical Analysis

R and R Studio were used for statistical analysis [34]. The frequency of occurrence of each code was quantified.

## 3 Results

Ninety‐four marketing assets were identified in Australia between 2018 and 2024, representing 20 campaigns for 13 ZAPs, manufactured by six alcohol companies. In NZ, 38 marketing assets were identified representing 10 campaigns for six ZAPs manufactured by three alcohol companies. Table S1 describes each included campaign by country, alcohol company and ZAP details, and how many assets were analysed for each campaign.

Broad categories of themes that were identified in the data included ‘occasion framing’, ‘benefits of consumption mentioned or implied’, ‘lifestyle, identity and value signifiers’ and ‘consumption cues’. Table 1 reports the proportion of campaigns assigned to each category and code, overall and by country. A fifth category of ‘price’ was also included, capturing strategies where the product was promoted in association with a price promotion (e.g., discount, value for money special or multibuy purchase) but no marketing assets were coded to this category.

In the first category, three quarters of campaigns (73%) suggested or showed the use of ZAPs in an established occasion, whereby the ZAP was seen as a replacement for an alcoholic product or was used in a traditional drinking occasion. Over half (53%) of campaigns suggested consumption in bars, pubs or other licenced venues. Almost two in three campaigns (63%) promoted alcohol‐branded ZAPs in the context of novel drinking occasions, that is, during occasions that expand current drinking contexts. All of these campaigns promoted context expansion via new drinking occasions such as on or around water (37%), while driving (36%) or during adrenaline or adventure‐type activities (30%, e.g., cliff jumping). Carlton Zero's *Rewrite the Rules* campaign was an example of a campaign creating additional drinking contexts; the protagonist was seen drinking the product while swimming, driving a go‐kart and operating heavy machinery during roadworks, all activities he noted he would not be able to do in an alcohol advertisement. Use of ZAPs while exercising or playing sport was also suggested in one in five campaigns (20%). In addition to the Carlton Zero campaign above, one further example of a campaign promoting ZAP use while exercising was Heineken's *Now you can* campaign, which showed a woman drinking the product as she stepped off a treadmill. Another was Gage Roads' *Longest Yeah Buoy Ever*, which showed a man doing a kettlebell swing with one hand while holding the ZAP in the other, and later riding a bike at the gym while drinking the product. Novel occasions promoting temporal expansion (27%, e.g., via ‘anytime’ or ‘weekday’ drinking) were seen in almost one in three campaigns, including the suggestion of ZAP use during abstinence periods (10%, e.g., Dry July) or while pregnant (3%). Just under half (47%) of the campaigns suggested ZAP use on established and novel occasions; 27% promoted use on only established occasions, while 17% promoted use on only novel occasions and 10% did not use occasion framing.

Almost all campaigns (93%) explicitly mentioned or showed, or implied, benefits of using a ZAP. The most frequently promoted benefit of ZAPs in campaigns was the choice of a viable alcohol alternative (73%), followed by taste (47%). Other noted benefits included fitness and health (27%, e.g., by promoting the use of the product as part of a healthy lifestyle or promoting exercise on the day after the drinking occasions), productivity or staying in control (23%, e.g., while at work, or during the day after a drinking occasion) and being a ‘better person’ (20%, e.g., being more present in relationships).

Across all identified campaigns, the most frequently identified lifestyle, identity and value signifier was mateship and friendship, with 80% of campaigns using this approach. Campaigns commonly used approaches of nature and conservation (50%) and humour (47%) to promote ZAPs. Half of all campaigns (50%) included an implicit or explicit consumption cue, with the most prevalent being use of the ZAP as a reward or for relaxation (43%).

## 4 Discussion

This novel research is the only study investigating the content of ZAP campaigns in the Australia and NZ markets, and adds to the very limited global evidence base. We found that while the majority of campaigns depicted ZAP use as a substitute for alcohol use or in contexts traditionally understood to be socially acceptable drinking occasions, a substantial proportion of campaigns (six in 10) promoted the ZAP in the context of non‐traditional drinking occasions, such as during exercise, while driving, or while operating heavy machinery. Our results are supported by previous research findings that ZAPs are presented as suitable for use across a wider range of occasions than alcoholic products [3], and that consumers interpret ZAP campaigns as providing cues that reinforce ZAPs as appropriate in times and contexts when alcohol use was not [2].

In the present study, the benefit of ZAPs appearing most frequently in campaigns was as a viable alternative to alcoholic products, potentially promoting inclusion in social occasions that have traditionally been associated with alcohol use. This positions ZAPs in settings where the social norm is that ‘everybody drinks’ [2]. The assessed campaigns also positioned ZAPs as a solution to some of the perceived disadvantages of alcohol use. In our sample, ZAPs were promoted as products to consume during a break at work, to be more present in relationships, while on a health kick, or to be able to exercise the next day. Similar reasons to drink ZAPs have been referenced by consumers in previous research, including benefits associated with productivity and control, for example, to feel ‘sharper’ the next day [7, 35]. One campaign encouraged use of the product during pregnancy with the tagline ‘Because … 9 months of moral support’. Concerns have previously been raised about the potential for zero and low alcohol products to mislead pregnant women about their alcohol content [1]. Our findings that benefits related to taste, health and product quality were prevalent is consistent with previous evidence that marketing of both alcoholic products and ZAPs promote these benefits [3].

Mateship or more broadly friendship was the most common lifestyle, identity or value signifier present in the sample of campaigns identified. Humour has been identified as a common approach in alcohol advertising in Australia, often combined with other prevalent themes such as mateship and friendship [33]. In the present study, the use of humour was apparent in almost half of all campaigns. Lifestyle, identity or value signifiers were communicated in overt or more implicit ways including the setting of the advertisement (e.g., in an urban environment or in nature) or by using language and imagery that evokes associations with desirable signifiers, thereby potentially building positive attitudes towards brands and products. When cues associated with nature are used to market alcoholic products, consumers may also believe that the product is ‘better for you’ [21]; this connection may also hold true with ZAP marketing.

Unlike previous studies of alcoholic product marketing demonstrating common marketing strategies of value for money and bulk purchases [33], we did not find evidence of price‐based strategies in our sample. One possible explanation is that ZAPs are often positioned (and priced) as premium ‘status goods’, rather than competing on price [36]. Had this sample included campaigns from alcohol retailers, who frequently engage in consumer‐facing price promotions [37, 38], these strategies may have been present.

We found that 40% of the campaigns were linked to sport as a lifestyle, identity or value signifier, with one in five demonstrating the use of ZAPs while exercising or playing sport. These findings are consistent with previous research positing that ZAP marketing may be part of a wider alcohol industry strategy to strategically align ZAP brands and products with sport, a common strategy used to promote alcohol brands and products [3, 39]. However, the results of the present study suggest that alcohol brands may utilise ZAPs to create opportunities in new sporting contexts that have not traditionally been associated with alcoholic beverages. For example, in 2024 the International Olympic Committee signed on their first ‘global beer sponsor’ AB InBev, led by zero alcohol product Corona Cero [40]. The campaign *For every golden moment* foregrounded this sponsorship. When young adults aged 18–24 in the United Kingdom were asked about the sponsorship, they suggested that consumers would perceive the sponsorship agreement and resultant campaign to be marketing the brand rather than the specific ZAP [41].

There were similarities between Australian and NZ campaigns in our findings, potentially indicating major alcohol companies are using similar strategies to promote ZAPs in these two countries. This similarity could be due to the global nature of the alcohol industry, which enables companies and brands to share resources, including budgets and marketing assets, across multiple regions [42].

### 4.1 Implications

While there is a need for governments and public health professionals to consider the potential benefits of the expanding ZAP market, findings of this research highlight that caution is warranted with respect to how alcohol‐branded ZAPs are advertised. The stated intent of alcohol advertising executives to compete with the soft drink market and expand opportunities for people to consume their products [7] was evident within the campaigns included in this research, with a substantial proportion promoting the use of ZAPs when alcoholic products would not normally be consumed. As previous research has identified, ZAPs can only provide a public health benefit if they replace alcoholic products [43]. We found evidence of ZAPs being promoted as alternatives to alcohol. This could be a benefit if promotion of ZAPs focussed on only replacing established drinking occasions. However, our findings also suggest that fewer than three in 10 campaigns only promote ZAPs as alternatives to alcohol. There are concerns about the alcohol industry's use of health messaging in ZAP marketing, particularly due to its contribution to a ‘halo effect’ whereby the industry appears to ‘do good’ but continues to contribute to harm from alcohol [44]. ZAPs are described by industry in public‐facing publications as tools to drive moderation, but in industry‐facing publications as tools to drive market growth [45]. The evidence that alcohol brands are competing with soft drinks and promoting the use of ZAPs in the context of new drinking occasions suggests brand strategies to expand the alcohol market, as opposed to an attempt to genuinely encourage consumers to replace alcoholic products with non‐alcoholic products. This approach appears to be working; alcohol industry market analyses have found that ZAPs are mostly replacing non‐alcoholic drinks such as soft drinks, water or hot drinks [46].

Given the similarities in the branding of ZAPs and alcoholic products from the same companies, health experts have expressed concern about the potential for ZAPs to create additional marketing opportunities [1, 36]. Alcohol industry analysts have previously identified that ZAP marketing provides different advertising opportunities with fewer restrictions on marketing compared to alcoholic products [46]. If alcohol‐branded ZAP marketing is being used to circumvent already weak controls on alcohol advertising, this is problematic from a public health policy perspective. This is especially relevant in light of recent research suggesting that children and young people are exposed to ZAP advertising, in part due to their wide availability in settings frequented by children (e.g., supermarkets), and that this exposure to ZAPs prompts them to think of alcohol [10, 47]. Given the emerging and well‐established concerns, a policy option available to Australian and NZ governments is to implement a comprehensive alcohol marketing regulatory framework with a legislative basis that prioritises community safeguards and includes marketing of ZAPs that share branding with alcoholic products. This should include appropriate safeguards for promotions in association with pregnancy.

### 4.2 Strengths and Limitations of the Study

The primary limitation of this study relates to the representativeness of the sample. Only publicly available marketing assets were included and analysed; it is possible that additional marketing assets (e.g., targeted campaigns on some social media platforms) were not identified. The scope of the study excluded ZAPs that do not share branding with an alcoholic product and campaigns without videos, meaning that results may not be generalisable to other campaigns or promotions, especially given the small sample size in each country. The primary strength of this study was the use of a systematic approach to identifying alcohol companies that own ZAPs and their marketing assets, resulting in an exhaustive product list across the two countries. The inclusion of mass‐media, video‐led campaigns is likely to have captured campaigns with substantial budgets that would have reached wide audiences, and therefore may be reflective of the exposure of large populations.

### 4.3 Future Research

The range of campaigns identified here provides a useful basis for future studies that investigate potential consumers' perceptions of common marketing approaches. In particular, future research could investigate the impact of ZAP marketing on specific population subgroups, including children, young people, people with a lived or living experience of alcohol dependence, pregnant people and First Nations peoples. Monitoring industry developments and marketing activities will be important to help inform policy responses, as will assessing compliance of campaigns against existing codes. Consideration could also be given to how new‐to‐world ZAP brands have positioned themselves and the strategies used to promote these products in comparison to how alcohol‐branded ZAPs are promoted.

## 5 Conclusions

Established alcohol brands appear to be using zero alcohol product marketing campaigns to promote alcohol‐branded products in new drinking contexts. This research adds to the existing limited international literature that explores how ZAPs are promoted. We call for strengthened community protections for alcohol marketing, including marketing by zero alcohol products that share branding with alcoholic products.

## Author Contributions

Each author certifies that their contribution to this work meets the standards of the International Committee of Medical Journal Editors.

## Funding

This research was supported by a Western Australian Government Mental Health Commission service agreement (MHC778) and a Healthway service agreement (G‐202403‐86909) for projects to support reducing harm from alcohol. A.B. receives funding from the Australian Department of Health, Disability and Ageing to support research regarding alcohol and other drugs. S.P. is funded by a National Health and Medical Research Council Investigator Grant (APP2034602).

## Conflicts of Interest

N.J.H. is a Guest Editor for the ‘Understanding the emergence, availability and consumption trends of no and low alcohol (“NoLos”) beverage products’ Special Section in *Drug and Alcohol Review*. They were excluded from all editorial decision‐making related to this manuscript. The other authors declare no conflicts of interest.

## Supporting information


**Table S1:** Alcohol‐branded zero alcohol product marketing campaigns in Australia and Aotearoa New Zealand (NZ).
**Table S2:** Data dictionary.

## Data Availability

The data that support the findings of this study are available from the corresponding author upon reasonable request.
