# Supplementary material for: Brand Sharing and New Drinking Occasions: A Content Analysis of How Alcohol Brands in Australia and Aotearoa New Zealand Promote Zero Alcohol Products
Source: Drug Alcohol Rev. 2026 Apr 13;45(4):e70148. doi: 10.1111/dar.70148 (PMC13075623; doi:10.1111/dar.70148)
Supplement: Supplementary file 1 — Table S1: Alcohol‐branded zero alcohol product marketing campaigns in Australia and Aotearoa New Zealand (NZ). Table S2: Data dictionary. [file DAR-45-0-s001.docx]

**Table S1.** Alcohol-branded zero alcohol product marketing campaigns in Australia and Aotearoa New Zealand (NZ).

| **Alcohol company** | **Zero alcohol product** | **Marketing campaign title** | **Launch date** | **Channels** | **Number of marketing assets^1^** |
| --- | --- | --- | --- | --- | --- |
| *Australian sample* | | | | | |
| Asahi Beverages | 4 Pines Ultra Low | If you love something, you don’t have to let it go | June 2022 | Digital, social media | 9 |
|  | Asahi Super Dry 0.0 | Beyond Expected | May 2023 | TV, video on demand, out of home, digital and social media | 4 |
|  | Carlton Zero | Rewrite the Rules | December 2020 | TV, out of home, radio, trade, and social media | 5 |
|  |  | Zero to Hero | May 2019 | TV, radio, digital | 3 |
|  | Corona Cero | For Every Golden Moment | June 2024 | Product packaging, films, social media, out of home, trade | 2 |
|  | Peroni Nastro Azzuro 0.0 | Scuderia Ferrari | January 2024 | Digital and social media | 3 |
|  |  | Back to Zero | July 2022 | TV, video on demand, out of home, digital, and social | 1 |
| Australian Vintage Limited | McGuigan Zero | Just Because | July 2021 | Digital and out of home | 21 |
|  |  | Zero Explanations Handbook | October 2021 | Digital and social | 4 |
| Diageo | Gordon’s 0.0 | Many Evenings In | January 2024 | TV, video on demand, social and digital | 1 |
| Gage Roads Brewing | Yeah Buoy | Longest Yeah Buoy Ever | July 2022 | Social media | 4 |
| Kirin | Guinness 0.0 | Lovely Day for a Guinness 0.0 | March 2024 | Social, digital, out of home | 1 |
|  |  | Now You Can | September 2019 | TV, social, digital, out of home, print, trade | 12 |
|  | Heineken 0.0 | Cheers with no alcohol. Now you can | January 2022 | TV, social, digital, out of home | 1 |
|  |  | The Best Driver | July 2023 | Digital, social, out of home | 7 |
|  |  | Sounds Like Good Times with F1 | March 2024 | TV, digital, social, out of home, radio | 6 |
|  |  | Player 0.0 | March 2023 | Digital, social, out of home | 3 |
|  | James Squire Zero | Full flavour, zero alcohol | May 2022 | Out of home, radio, social | 2 |
|  | XXXX Zero | Give a XXXX about tomorrow | August 2022 | TV, out of home, broadcast video on demand, digital, social | 2 |
| Treasury Wine Estates | Wolf Blass Zero | Why settle when you can soar | August 2022 | TV, out of home, broadcast video on demand, digital, social | 3 |
| *NZ sample* | | | | | |
| Lion NZ | Steinlager Alcohol Free | Here’s to tomorrows | February 2022 | Social, print | 8 |
|  | Zeffer 0% Crisp Apple Cider | Real Hawke’s Bay cider and seltzer | January 2021 | Video on demand | 5 |
|  |  | 100% great taste. 0% alcohol. | November 2022 | Video on demand | 2 |
| DB Breweries | DB Export Gold 0.0% | That’s Why | March 2020 | TV, out of home, radio | 3 |
|  | Heineken | Beyond Zero | May 2023 | Video on demand, social | 8 |
|  |  | Enjoy a beer, not a compromise | June 2022 | Out of home, social | 4 |
|  |  | Now you can | August 2018 | TV, digital | 3 |
| Asahi Brewing | Peroni 0.0 | Live every moment | August 2022 |  | 1 |
|  | Asahi Super Dry 0.0% | Beyond expected | May 2023 | Digital, out of home, on-premises | 1 |
|  |  | Rugby World Cup 2023 | September 2023 | Digital, social | 3 |

^1^ We considered marketing assets to be unique executions of campaigns, including unique executions on the same channel (e.g., print ads with different imagery and/or taglines) or either unique or similar executions (same/similar imagery and taglines) on different channels (e.g., a 30-sec television video and a cut-down 15-sec social media version); each of these examples was considered a marketing asset.

**Table S2.** Data dictionary

| **Category** | **Marketing message code** | **Code definition** |
| --- | --- | --- |
| **Occasion framing** | **Established occasions** | Suggestion, implied or perceived, to replace an alcoholic product with a zero alcohol product, to use zero alcohol at times when the consumer would typically drink an alcoholic product. |
|  | **Established occasions: In bar or pub** | The product is shown consumed in the context of a traditional drinking environment such as a pub, bar or other licensed venue. |
|  | **Established occasions: Partying** | Product is portrayed as being used during a party, among groups of people at a licensed venue such as a bar, etc. |
|  | **Established occasions: Special occasion** | Consume the product on a special occasion such as a birthday celebration, important gathering. |
|  | **Novel occasions** | Creation of new moments to drink zero alcohol products, particularly in situations and on occasions in which alcoholic products are generally not consumed, or among people who don't drink alcohol. |
|  | **Novel occasions: context expansion** | Consume zero alcohol in new locations where alcohol consumption is not common or is socially unacceptable. |
|  | **Novel occasions: context expansion: Exercising or playing sport** | Consume the product while doing sport or exercise. |
|  | **Novel occasions: context expansion: On or around water** | Product shown (or shown to be used) in or around water environments, including a beach, river, pool, lake or another waterway. |
|  | **Novel occasions: context expansion: Driving** | Consume the product while driving, or imply that it would be appropriate/safe to drive after consumption of the product. |
|  | **Novel occasions: context expansion: Adrenaline activities** | The product shown in association with adrenaline activities such as mountain climbing, water skiing, driving a speedboat |
|  | **Novel occasions: context expansion: Work** | Promotion of the product in the context of a work environment, for example during a job interview, a lunch break, while operating heavy machinery at work. |
|  | **Novel occasions: temporal expansion** | Encourage consumption at times when alcohol may not typically be consumed (e.g., before 'big day' or 'fresh week' at work or anytime of day) |
|  | **Novel occasions: temporal expansion: Abstinence periods** | Promotion of the product in association with a short-term period of abstinence such as Dry January, Dry July, Ocsober etc. |
|  | **Novel occasions: temporal expansion: While pregnant** | Encouraging consumption of the product among people who are pregnant or planning pregnancy. |
| **Benefits of consumption** | **Productivity / control** | Implied benefits of use of product include productivity, being able to take control at work and other life events, being present at work/life, having a clear head before a 'fresh week' at work, to be productive, avoiding a hangover, and similar messaging. |
|  | **Choice of viable alcohol alternative** | The product offers consumers the choice to consume a viable alternative to alcohol. The product can help consumers feel included in social situations. |
|  | **Being a ‘better’ person** | The product helps consumers be a 'better person' (e.g., by being present at life events or in important relationship, being a better parent/sibling/partner/friend/child), helps improve wellbeing by helping them to avoid alcohol use, ad portrays the wellbeing benefits of not drinking alcohol or of drinking the product being promoted. |
|  | **Perceived health benefits** | Product is advertised in a way that positions it as a 'healthier' alternative to alcohol, for example promoting reduced calories as a 'health benefit', suggesting the product's use is compatible with and/or could be incorporated into exercise regimes and sporting activities, positioning the product alongside healthier foods such as salads, positioning the product as part of a healthy lifestyle through linking to sport and gym. |
|  | **Taste / refreshing** | Mention of taste/flavour (e.g., full flavour, tastes just like beer, etc.). Mention of the product being refreshing or quenching thirst. Campaign material portrays the product as refreshing or thirst-quenching, or satisfying. |
|  | **Quality product** | Promote the use of quality ingredients or quality processes undertaken to manufacture the product. Discussion of/focus on particular ingredients in the advertising materials. |
| **Lifestyle, identity and value signifiers** | **Sport** | Linking the product to sporting events, sportspeople, sport sponsorship, or use of sporting imagery, language and/or metaphors within the marketing materials. |
|  | **Masculinity / Manliness** | Promoting the product in the context of or in association with manliness and masculinity, traditionally male roles and concepts. |
|  | **Femininity / gender** | Promoting the product in the context of or in association with femininity and female gender, conventional and normative female roles and concepts. Use of pink, mummy wine culture, and other feminine identity markers to promote the product. |
|  | **Nature / conservation** | Promotion of product in association with nature, floral or nature-related imagery, promotion of the 'naturalness' of a product, 'natural' ingredients, conservation, botanical language and imagery, gardening, nature colour palettes (greens, browns, etc), envrionmentally conscious. |
|  | **Urban lifestyle** | Linking the product to urban traditional drinking environments, such as restaurants, bars, or promoting the product in association with food and other drinks. |
|  | **Exclusivity and expertise** | Positioning of the product as being of a certain status, luxury/upmarket, or associated with drinkers who are 'cool and hipster'. Promotion of product as being for affluent consumers with particular tastes, implications of exclusivity through marketing by use of phrases such as 'world's first', 'limited edition', 'sneak peak', and 'secret' events. Positioning the product or the brand as being knowledgeable or having expertise/experience. |
|  | **Heritage** | Promotion of the brand or product heritage. |
|  | **Future** | Appeals to the values of people who are environmentally conscious, those who are thinking about the future of the planet, and the impact that alcohol is having on their health and on the world; new technology for removing alcohol content |
|  | **Humour** | Use of humour to promote the product, marketing material appeals to people's sense of humour. |
|  | **Mateship / friendship** | Promotion of the product portraying groups of friends and/or using values associated with friendships and mateship |
|  | **Family** | Family values, families, child/parent, sibling relationships portrayed in the advertising materials. |
|  | **First Nations peoples** | Use of First Nations peoples, iconography, or imagery. Includes Aboriginal, Torres Strait Islander, and Māori peoples and communities. |
|  | **Sexual attraction** | Use of sexual imagery in the advertising, material implies sexual benefits from use of the product, material implies or shows sexual successes associated with the product. |
| **Consumption cues and guidelines** | **Reward / break / relaxing** | Consume the product as a reward, while taking a break, or to relax. |
|  | **How to drink / cocktails / recipes** | Promotion of the product in association with suggestions of drink recipes or ideas and suggestions of how to drink and serve the product. |
|  | **Drinking to excess / drinking inconsistent with community standards** | Depicting the product in a way that is contrary to community standards on alcohol and health. For example, encouraging, directly or by implication, irresponsible or immoderate drinking. That applies to both the amount of product presented and the way drinking is portrayed. |
| **Price-based benefits** | **Value for money** | The product is promoted in association with a price promotion such as a discount or value for money special |
|  | **Buying in bulk** | Specials or discounts for purchasing the product a multibuy or in a pack |
